# Carbon Monoxide Alleviates Cardiomyocyte Pyroptosis in Diabetic Cardiomyopathy by Downregulating the IL‐33/ST2L Axis

**DOI:** 10.1002/iid3.70231

**Published:** 2025-08-23

**Authors:** Chunjie Jiang, Ping Zhu, Ping Yao, Xiaojun Bi, Chao Gao

**Affiliations:** ^1^ Jinan University Affiliated Guangzhou Red Cross Hospital Guangzhou China; ^2^ Department of Nutrition and Food Hygiene, School of Public Health, Tongji Medical College Huazhong University of Science and Technology Wuhan China; ^3^ Department of Medical Ultrasound, Tongji Hospital, Tongji Medical College Huazhong University of Science and Technology Wuhan China; ^4^ Key Laboratory of Trace Element Nutrition of National Health Commission, National Institute for Nutrition and Health Chinese Center for Disease Control and Prevention Beijing China

**Keywords:** carbon monoxide, diabetic cardiomyopathy, IL‐33/ST2L axis, pyroptosis

## Abstract

**Objectives:**

This study aimed to investigate whether carbon monoxide (CO) can alleviate cardiomyocyte pyroptosis by downregulating the IL‐33/ST2L axis in diabetic cardiomyopathy (DCM).

**Methods:**

The diabetic mouse model was established and treated with CO‐releasing molecule‐2 (CORM‐2) or invalid CORM‐2 (iCORM‐2). For in vitro studies, cardiomyocytes were treated with high glucose (HG).

**Results:**

The HG‐treated cardiomyocytes exhibited increased IL‐33, ST2L, and pyroptosis‐related protein expression compared with that in the control group (*p* < 0.05). Treatment with recombinant IL‐33 further increased the expression of HG‐induced pyroptosis‐related proteins (*p* < 0.05). Compared with control mice, DCM mice showed reduced cardiac function and elevated expression of IL‐33, ST2L, and pyroptosis‐related proteins (*p* < 0.01). Intervention with CORM‐2 ameliorated cardiac injury, and decreased the expression of IL‐33, ST2L, and pyroptosis‐related proteins in vivo and in vitro (*p* < 0.05). However, iCORM‐2 had no effect both in vivo and in vitro.

**Conclusions:**

In conclusion, CO may inhibit cardiomyocyte pyroptosis by downregulating the IL‐33/ST2L axis in DCM mice.

## Graphical abstract

1. IL‐33/ST2L axis upregulation can induce cardiomyocyte pyroptosis in diabetic cardiomyopathy. 2. Carbon monoxide alleviates heart injury in diabetic cardiomyopathy. 3. Carbon monoxide can reduce cardiomyocyte pyroptosis by inhibiting the IL‐33/ST2L axis in diabetic cardiomyopathy.

## 1 Introduction

The increased incidence of diabetes mellitus, especially type 2 diabetes mellitus (T2DM), and its associated complications have become a crucial public health concern worldwide [1, 2]. Diabetic cardiomyopathy (DCM) is a common cause of mortality in individuals with diabetes [3]. Epidemiological studies have demonstrated that over 50% of patients with diabetes exhibit some degree of myocardial injury [4]. DCM is primarily characterized by chronic cardiac inflammatory infiltration, fibrosis, and dysfunction [5]. Although it is widely recognized that chronic inflammation‐induced cardiomyocyte injury plays a crucial role in the progression of DCM, the underlying mechanisms remain unclear [6].

Pyroptosis is a programmed cell death process mediated by inflammasomes and cysteine‐aspartic acid‐specific protease 1 (caspase‐1) [7, 8]. During this process, gasdermin D (GSDMD) plays an important role in the formation of pores on the cell membrane surface, which promotes the release of inflammatory cytokines such as interleukin (IL)‐1β and IL‐18 by acting on cytosolic phosphatidylinositol [9]. Cardiomyocyte pyroptosis is a key pathological event in the progression of DCM, and inhibiting pyroptosis can ameliorate DCM injury [10, 11].

IL‐33 is an IL‐1 family cytokine that can activate intracellular signaling pathways by binding to its membrane receptor, the transmembrane growth stimulation expressed gene 2 (ST2L), and together they constitute the IL‐33/ST2L axis [12, 13]. The IL‐33/ST2 pathway is upregulated during heart failure, and this effect is increased under stress conditions [14]. In coxsackievirus B3‐induced mouse myocarditis, recombinant IL‐33 (rIL‐33) treatment increased the IL‐1β levels [15]. In addition, inflammatory injuries were observed in the hearts of normal mice following rIL‐33 administration, and IL‐33 administration increased cardiomyocyte death and overall mortality in the myocardial infarction mouse model [16]. Thus, IL‐33/ST2L axis upregulation in heart injury may promote inflammatory responses and cardiomyocyte death. However, whether it can induce pyroptosis remains unknown. Herein, we speculate that IL‐33/ST2L axis activation could induce cardiomyocyte pyroptosis in DCM.

Emerging evidence indicates that carbon monoxide (CO), a gaseous messenger molecule, possesses anti‐inflammatory properties [17, 18]. In vivo, CO is a by‐product of inducible heme oxygenase‐1 (HO‐1) [19]. Both endogenous CO/HO‐1 induction and exogenous CO‐releasing molecule (CORM) administration can alleviate organ and tissue inflammatory injuries. CO/HO‐1 attenuates mice asthma by reducing IL‐33 and inhibiting NLRP3 activation [20]. Similarly, CORM‐2 could reduce IL‐33 expression and exert protective roles in airway injuries and rheumatoid arthritis. Additionally, CORM‐3 has been found to reduce pyroptosis of hippocampal neurons, astrocytes, and amygdala neuronal cells in brain injuries [21, 22, 23]. In lipopolysaccharide (LPS)‐induced cardiac injury, CORM‐3 has been shown to attenuate the myocardial inflammatory response but had no effects on NLRP3^‐/‐^ mice [24]. However, it remains unclear whether or not CO can alleviate cardiomyocyte pyroptosis and attenuate DCM progression. In the present study, we aimed to investigate the effect of CORM‐2 on the IL‐33/ST2L axis in the context of focusing on cardiomyocyte pyroptosis in DCM.

## 2 Materials and Methods

### 2.1 Cell Culture and Treatment

HL‐1 cells were cultured in Dulbecco's Modified Eagle Medium (DMEM) (Gibco, USA) with 5.5 mmol (mM) glucose, supplemented with 10% fetal bovine serum (Gibco, USA) and 1% penicillin‐streptomycin antibiotic (Gibco, USA) in an incubator with 5% CO_2_ at 37°C. For inducing the cardiomyocyte glucotoxicity model, the cells were added and stimulated with 33 mM glucose. Cells cultured in DMEM with 5.5 mM glucose plus 27.5 mM mannitol were collected as controls.

### 2.2 Experimental Animals and Treatments

Male C57BL/6J mice aged 8 weeks were randomly assigned to either a normal diet group or a high‐fat diet (HFD, 60% of energy from fat) group. After 4 months, the HFD mice were intraperitoneally injected with streptozotocin for five consecutive days (50 mg/kg, Sigma‐Aldrich, USA) as previously described [25]. Mice with blood glucose levels above 16.7 mmol/L were considered diabetic. The blood glucose was measured by Accu‐Chek Performa glucometer (Roche Diagnostics, Mannheim, Germany). Subsequently, mice were divided into four groups (*n* = 12): a control group (Ct), a diabetic cardiomyopathy group (DCM), a DCM + CORM‐2 group (DCM + CORM‐2), and a DCM + inactive CORM‐2 group (DCM + iCORM‐2). After the grouping process, diabetic mice underwent a 4‐month intervention with CORM‐2 and iCORM‐2. CORM‐2 (3 mg/kg = 5.86 μmol/kg, twice weekly, Sigma‐Aldrich, USA) and iCORM‐2, which is produced by releasing CO from CORM‐2 at room temperature, were administered intraperitoneally. After 3 days of the last administration, heart tissue and serum samples were collected from the mice for experimental analysis. The iCORM‐2 was prepared by incubating CORM‐2 dissolved in DMSO for 24 h at room temperature to liberate CO and then blowing nitrogen gas to exhaust CO. After 3 days of the last administration, cardiac tissue and serum samples were collected from the mice for experimental analysis. All diets were purchased from Medicience (Yangzhou, China). All animal procedures were carried out according to the ‘Guiding Principles in the Care and Use of Animals approved by the Tongji Medical College Council on Animal Care Committee’. The approved number is [2017] S820.

### 2.3 LDH Release

LDH activity in the cell culture medium (supernatant) was detected as previously described [26]. At the end of the treatments, cytosolic supernatants were collected and their optical density was measured at 450 nm according to the instructions (Beyotime Biotechnology, China). The results were expressed as 100 × (experimental LDH − spontaneous LDH)/(maximum LDH release − spontaneous LDH).

### 2.4 Cell Counting Kit‐8 (CCK‐8) Assay

Cells were seeded into 96‐well cell culture plates. After treatment, 10 µL CCK‐8 solution (Beyotime Biotechnology, China) was added and incubated at 37°C for 1 h. The optical density was measured at 450 nm according to the instructions.

### 2.5 Immunofluorescence

After culture and treatment, the cells were washed with PBS, then fixed in 4% paraformaldehyde and permeabilized with 0.2% Triton‐100 for 15 min. Next, cells were incubated with primary antibodies (IL‐33, 12372‐1‐AP, Proteintech; ST2, 11920‐1‐AP, Proteintech) at 4°C overnight, followed by incubation with secondary antibody (Alexa Fluro 488 goat anti‐rabbit IgG) for 2 h and DAPI staining solution (Beyotime) for 5 min at room temperature. Anti‐fluorescence quenching agent was added and the cells were photographed using a fluorescence microscope (MShot Image Analysis System).

### 2.6 Western blot

Heart tissues or HL‐1 cells were homogenized and lysed with Radio‐Immunoprecipitation Assay Lysis Buffer for 30 min. The protein concentrations in the lysates were then quantified using a BCA protein assay kit (Beyotime Biotechnology). Immunoblotting was performed as per the manufacturer's guidelines (Bio‐Rad, Hercules, CA). The membranes were blocked with a washing buffer containing 5% skimmed milk for 2 h. Then each target band was incubated overnight at 4°C with the specific primary antibody and 1 h at room temperature with the corresponding secondary antibody (goat HRP‐linked anti‐rabbit/mouse IgG, CST). Specific primary antibodies are anti‐NLRP3 (19771‐1‐AP, Proteintech), anti‐GSDMD (A10164, ABclonal) anti‐caspase‐1 (Santa Cruz, USA), anti‐IL‐1β (16806‐1‐AP, Proteintech), anti‐IL‐33 (12372‐1‐AP, Proteintech), anti‐ST2 (11920‐1‐AP, Proteintech) and anti‐GAPDH (60004‐1‐Ig, Proteintech). Optical densities of the membranes were quantified with Image J software. Immunoblotting was performed as previously described [27].

### 2.7 Staining of Tissue Sections

Hearts were dissected, samples of left ventricular myocardium were removed and put into 4% paraformaldehyde for 48 h and slightly trimmed to make myocardial tissues close to block shape. Then the cardiac samples were embedded in paraffin and sliced into histological sections (7 μm). H&E staining was performed to analyze the general architecture of the cardiac muscle. Masson trichrome staining and Sirius‐red staining were performed to analyze cardiac fibrosis. Immunohistochemistry staining was performed to observe the expression level of IL‐33 (ab54385, Abcam) and ST2 (ab25877, Abcam) in myocardial sections. The stained sections were observed under a digital image microscope (Olympus, Tokyo, Japan).

### 2.8 Echocardiography

At the end of the CO intervention, the M‐mode and B‐mode of the mice were measured using a Vevo 2100 system (VisualSonics, USA) in the department of medical ultrasound, Tongji Hospital of Huazhong University of Science and Technology.

### 2.9 Transmission Electron Microscope (TEM)

Heart tissues were cut into 1 mm strips and then fixed in glutaraldehyde (2.5%), and then processed as previously described [28]. The images were observed using TEM (FEI Tecnai G2 20, USA).

### 2.10 ELISA Assay

ELISA kits for IL‐33 (15105‐s, mlbio) and sST2 (MM‐1070M1, MEIMIAN) were obtained and the tests were performed following the manufacturer's instructions. The optical density was observed using a SYNGENE Multifunctional microplate reader (BioTek).

### 2.11 Statistical Analysis

Data were analyzed using GraphPad Prism 9 and presented as mean ± SEM. Student's *t*‐test was used to compare data from two groups. Comparisons between more than two groups were assessed by one‐way ANOVA with Turkey's analysis. *p* < 0.05 was set as a significant difference.

## 3 Results

### 3.1 High Glucose Treatment Induced Cardiomyocyte Pyroptosis

**Figure 1:**
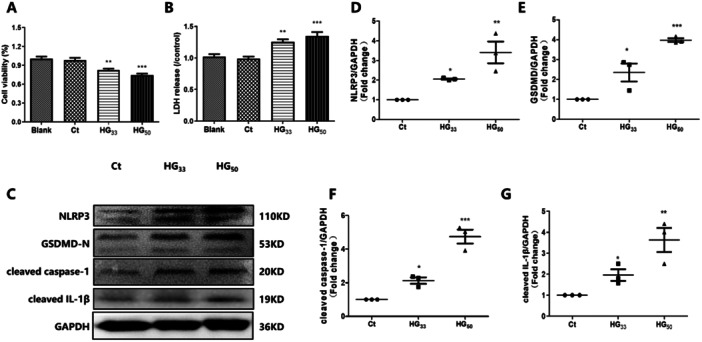
HG treatment induced HL‐1 cells pyroptosis. (A) CCK‐8 was performed to measure the cell viability (*n* = 5). (B) LDH release in the culture medium of HL‐1 cells (*n* = 5). (C–G) Western blot was performed to measure the expression of pyroptosis‐related proteins including NLRP3, GSDMD‐N, cleaved caspase‐1, and cleaved IL‐1β (*n* = 3). *p < 0.05 vs. the Ct; **p < 0.01 vs. Ct; ***p < 0.001 vs Ct. The results are presented as the mean ± SEM. Blank: 5.5 mM glucose; Ct: control, 5.5 mM glucose plus 27.5 mM mannitol; HG_33_, 33 mM glucose; HG_50_, 50 mM glucose.

To examine the effect of high glucose (HG, 33 mM) on HL‐1 cells, we first evaluated cell viability. After 48 h of HG treatment, we observed a decrease in the cell viability (Figure 1A, *p* = 0.002) and an increase in LDH release (Figure 1B, *p* = 0.023) in the HG group compared with the control. Moreover, the expression of pyroptosis‐related proteins including NLRP3, GSDMD‐N, cleaved caspase‐1, and cleaved IL‐1β, was also increased after HG treatment (*p* < 0.05, Figure 1C–G). Additionally, exposure of HL‐1 cells to high glucose (HG) at a concentration of 50 mM led to a further reduction in cell viability, an additional elevation in LDH release, and an enhanced upregulation of pyroptosis‐related protein expression. These results indicate that HG treatment promotes the pyroptosis phenotype in the HL‐1 cells.

### 3.2 The IL‐33/ST2L Axis Aggravated the Pyroptosis in the HG‐Incubated HL‐1 Cells

**Figure 2:**
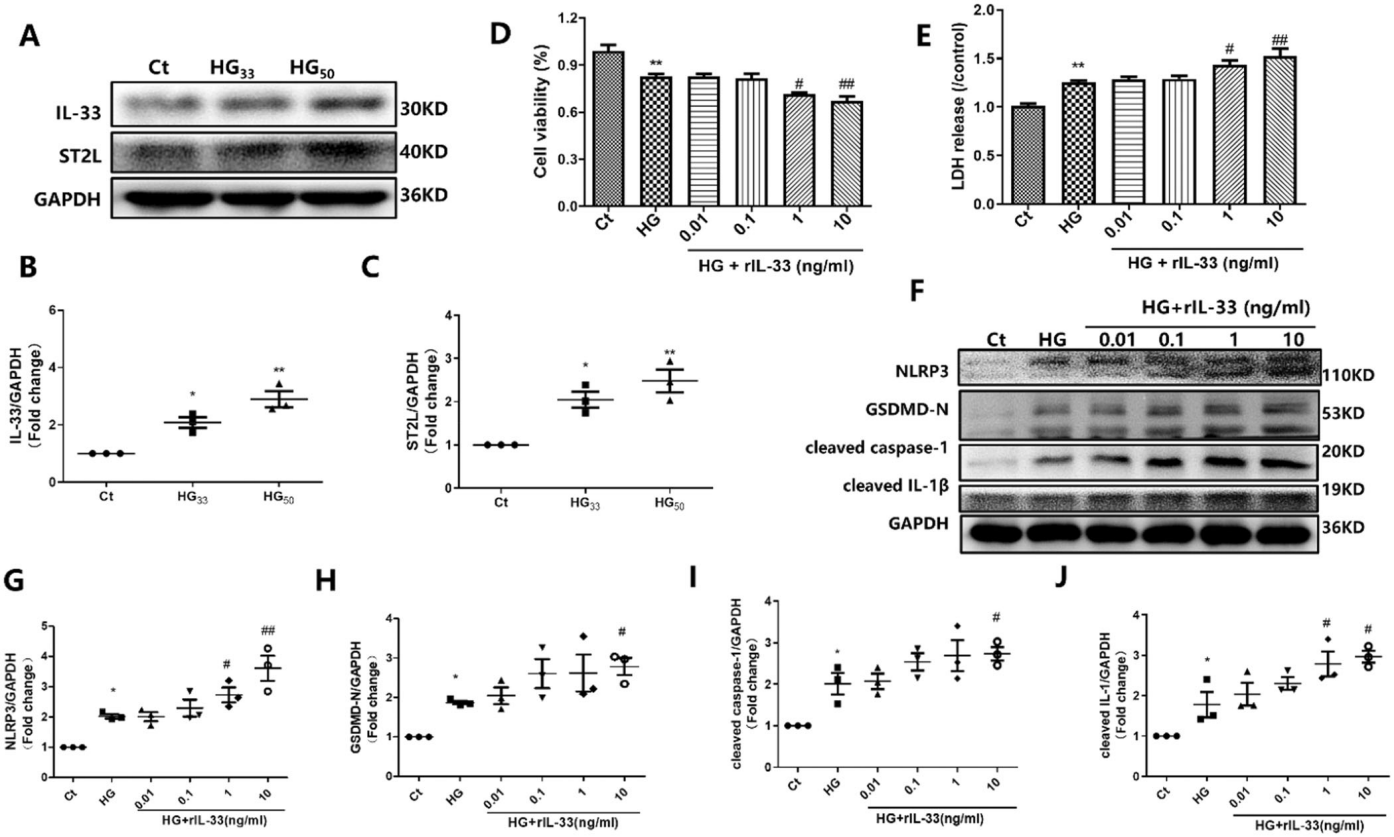
The rIL‐33 treatment exacerbated HG‐induced pyroptosis in HL‐1 cells. (A–C) Western blot was performed to measure the expression of IL‐33 and ST2L (*n* = 3). (D) CCK‐8 was performed to measure the cell viability (*n* = 5). (E) LDH release in the culture medium of HL‐1 cells (*n* = 5). (F–J) Western blot was performed to measure the expression of pyroptosis‐related proteins including NLRP3, GSDMD‐N, cleaved caspase‐1 and cleaved IL‐1β (*n* = 3). Ct, control; HG, high glucose. *p < 0.05 vs. the Ct; **p < 0.01 vs. Ct. ^#^ *p* < 0.05 vs. HG; ^##^ *p* < 0.01 vs. the HG. The results are presented as the mean ± SEM.

Owing to the potentially important role of the IL‐33/ST2L axis in heart failure, we examined this axis with respect to pyroptosis. We evaluated the expression of IL‐33 and its membrane receptor ST2L in HG‐incubated HL‐1 cells. After 48‐h HG treatment in HL‐1 cells, IL‐33 and ST2L expression was increased (*p* = 0.027 and *p* = 0.033) (Figure 2A–C). Next, we treated HG‐incubated HL‐1 cells with rIL‐33 for 48 h. As shown in Figure 2D–E, compared with the HG group, 1 ng/mL rIL‐33 treatment increased LDH release (*p* = 0.023), decreased cell viability (*p* = 0.041), and increased NLRP3 and IL‐1β protein levels (*p* = 0.034 and *p* = 0.047). Furthermore, 10 ng/mL rIL‐33 treatment exacerbated HL‐1 cell damage and significantly increased NLRP3, GSDMD‐N, cleaved caspase‐1, and cleaved IL‐1β expression (*p* < 0.05) (Figure 2F–J). These results suggest that IL‐33 exacerbated HG‐induced pyroptosis in HL‐1 cells.

### 3.3 Carbon Monoxide (CO) Improved Pyroptosis Markers in HG‐Treated HL‐1 Cells

**Figure 3:**
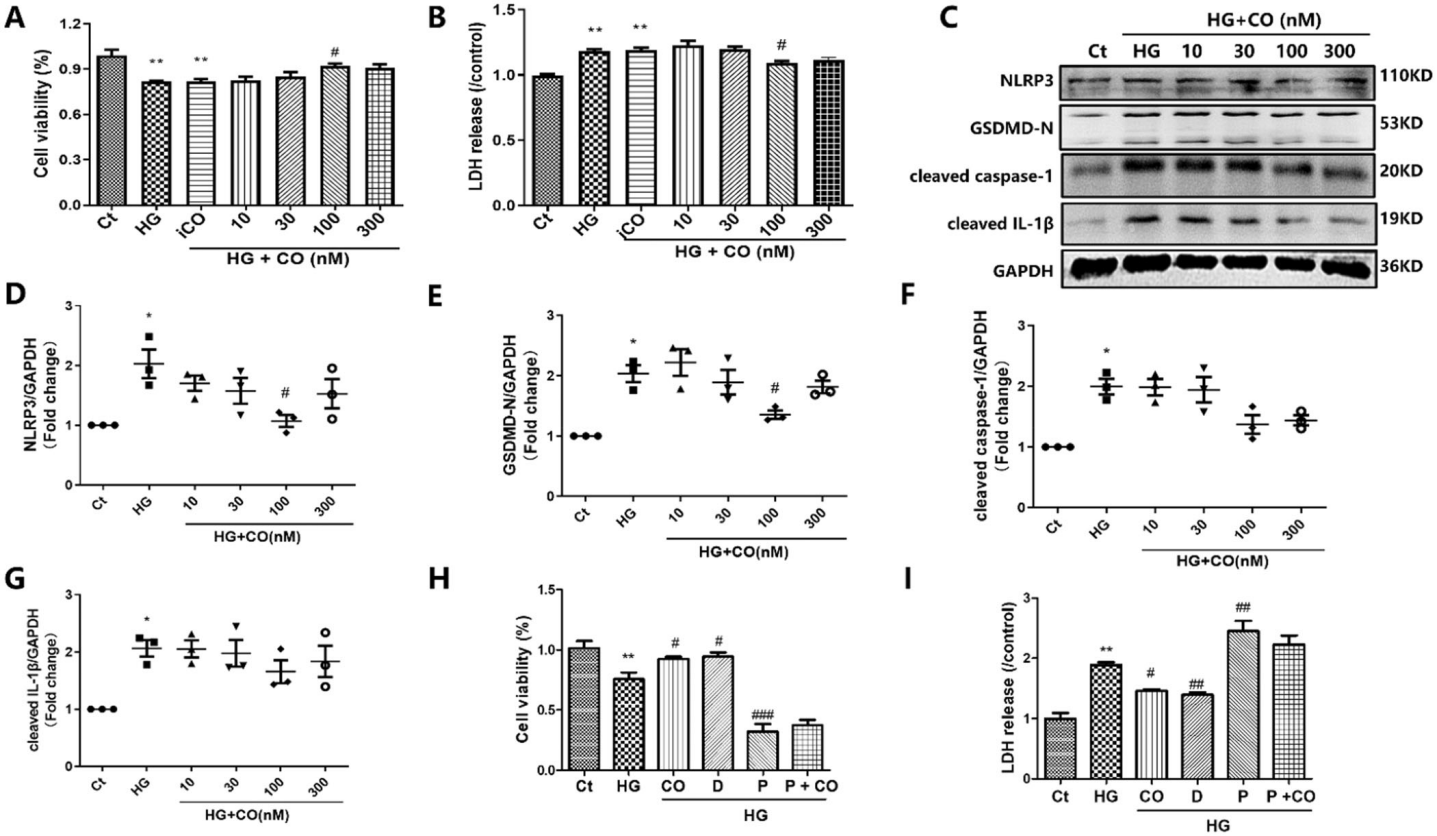
CORM‐2 treatment alleviated HG‐induced pyroptosis in HL‐1 cells. (A) CCK‐8 was performed to measure the cell viability (*n* = 5). (B) LDH release in the culture medium of HL‐1 cells (*n* = 5). (C–G) Western blot was performed to measure the expression of pyroptosis‐related proteins including NLRP3, GSDMD‐N, cleaved caspase‐1 and cleaved IL‐1β (*n* = 3). (H–I) The effects of CORM‐2 (100 nM), pyroptosis inhibitor disulfiram, and pyroptosis inducer polyphyllin VI on the LDH release and cell viability of HG‐incubated HL‐1 cells (*n* = 5); Ct, control; HG, high glucose. *p < 0.05 vs. the Ct; **p < 0.01 vs. the Ct; ^#^ *p* < 0.05 vs. the HG; ^##^ *p* < 0.01 vs. the HG. The results are presented as the mean ± SEM. D: Disulfiram (10 μM), P: Polyphyllin Ⅵ (5 μM).

CO demonstrates anti‐inflammatory activity. Thus, HG‐incubated HL‐1 cells were treated with CORM‐2. As shown in Figure 3A,B, CORM‐2 (100 nM) treatment ameliorated both the decreased cell viability (*p* = 0.032) and the increased LDH release (*p* = 0.028) observed after the 48 h HG treatment, whereas iCORM‐2 (100 nM) treatment showed no effect. Additionally, CORM‐2 significantly decreased NLRP3 and GSDMD‐N (*p* < 0.05) expression. Subsequently, we treated HG‐incubated HL‐1 cells with pyroptosis inhibitor disulfiram. CORM‐2 and disulfiram showed similar effects in improving cell viability and decreasing LDH release in HG‐incubated HL‐1 cells (Figure 3H–I). In line with this observation, the pyroptosis activator polyphyllin VI significantly decreased cell viability and elevated LDH levels, whereas CO treatment led to a slight decrease in LDH. However, this difference was not statistically significant.

### 3.4 CORM‐2 Reduced IL‐33 and ST2L Expression in HG‐Incubated HL‐1 Cells

**Figure 4:**
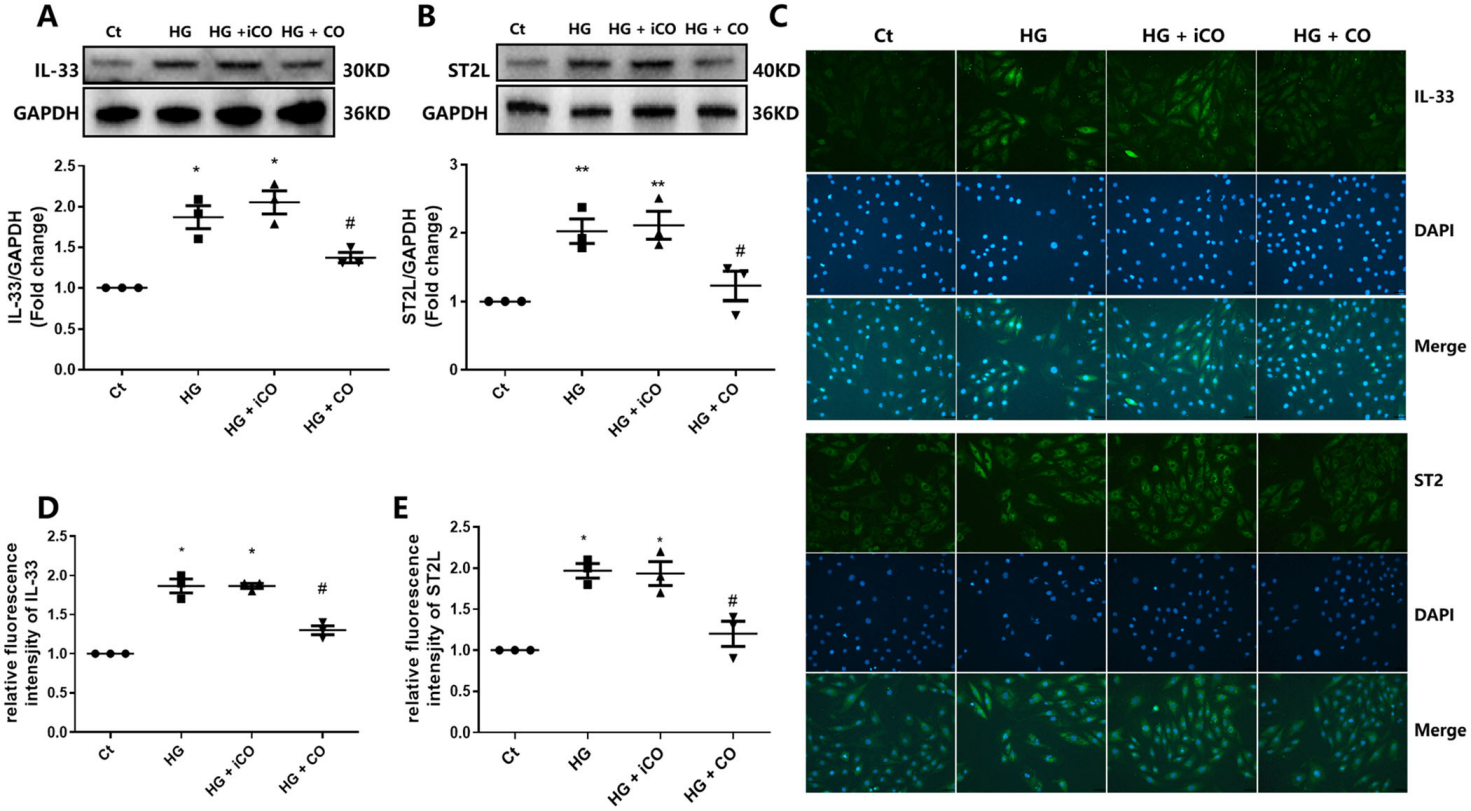
CORM‐2 treatment decreased the expression of IL‐33 and ST2L in HG‐incubated HL‐1 cells. (A and B) Western blot was performed to measure the protein expression of IL‐33 and ST2L in HG‐incubated HL‐1 cells. (C–E) Representative microscopy images and the relative immunofluorescence intensity of IL‐33 and ST2L in HL‐1 cells (200×). Ct, control; HG, high glucose. *p < 0.05 vs. the Ct; **p < 0.01 vs. the Ct; ^#^ *p* < 0.05 vs. the HG. The results are presented as the mean ± SEM (*n* = 3).

We demonstrated that IL‐33 exacerbates pyroptosis in HG‐treated cells, and CO was reported to have the potential to decrease IL‐33 [20]. Therefore, we evaluated the protein expression of IL‐33 and ST2L in CORM‐2‐treated HL‐1 cells. As shown in Figure 4A,B, a 48‐h CORM‐2 treatment decreased both IL‐33 and ST2L protein expression in HG‐incubated HL‐1 cells. In agreement with the Western blot results, immunofluorescence also revealed that the relative intensity of IL‐33 and ST2L was decreased after CORM‐2 treatment. However, iCORM‐2 treatment did not affect the expression of either IL‐33 and ST2L (Figure 4C–E).

### 3.5 CORM‐2 Alleviated Heart Injury in DCM Mice

**Figure 5:**
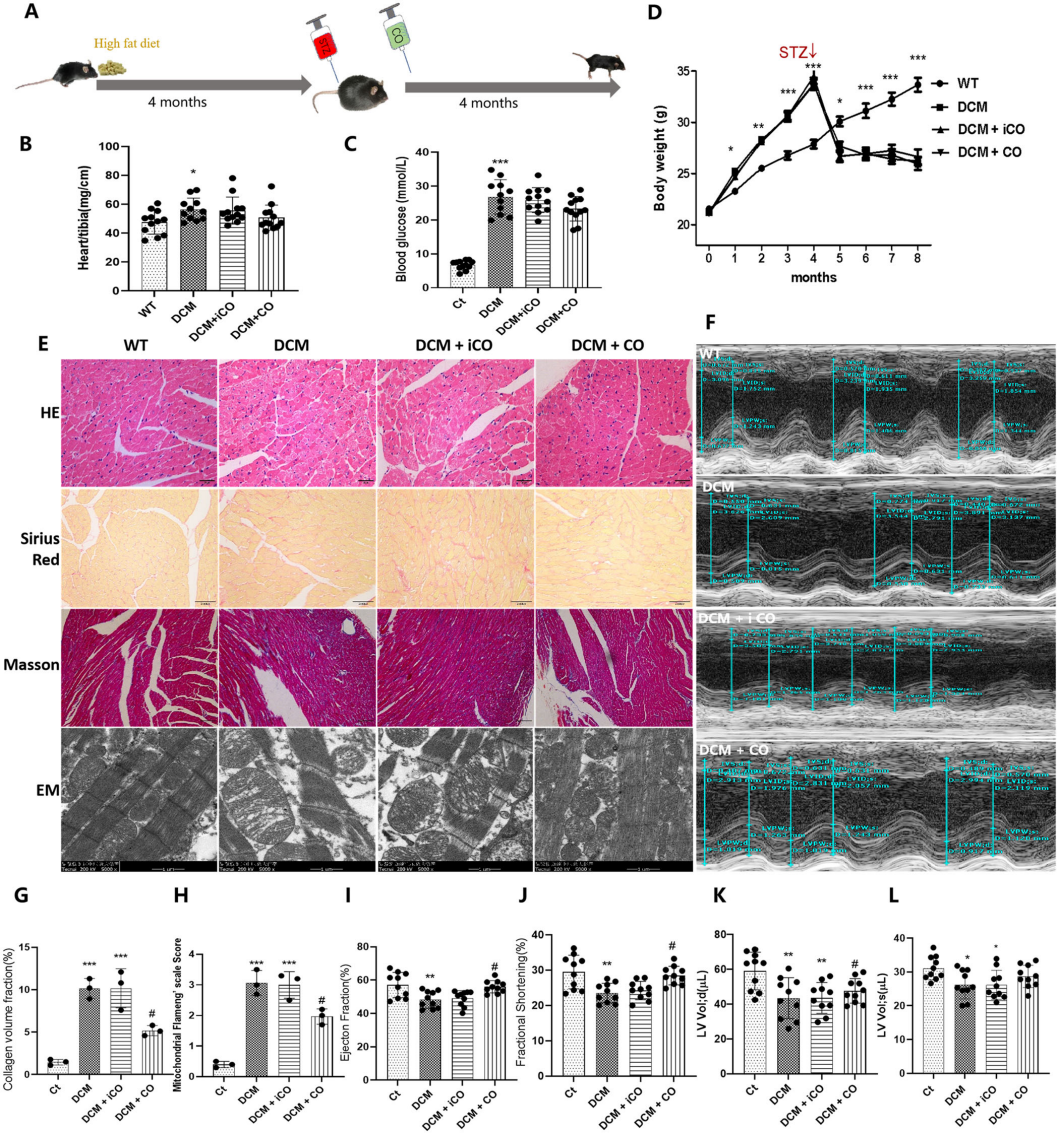
CORM‐2 treatment alleviated heart injury of mice with DCM. (A) Experimental timeline for the development and intervention of the mouse model. (B) The ratio of heart weight to tibia length. (C) The blood glucose was tested with the glucose oxidase‐peroxidase method. (D) The body weight changes of the mice (*n* = 11–12). (E) Representative images of heart tissues stained with HE, Sirius red, Masson trichrome, and ultra‐structural changes in mitochondria and myofilaments assessed by transmission electron microscopy. (F) Representative pictures of M‐mode echocardiography. (G) The analysis of collagen volume fraction in the images of heart tissues stained with Masson trichrome (*n* = 3). (H) Statistical results of mitochondrial Flameng score in different groups (*n* = 3). (I–L) The analysis of fractional shortening, ejection fraction, left ventricular end‐systolic volume (LV Vol; d), and left ventricular end‐diastolic volume (LV Vol; d) in the cardiac function of mice (*n* = 10). *p < 0.05 vs. the Ct; **p < 0.01 vs. Ct; ***p < 0.001 vs. Ct; ^#^ *p* < 0.05 vs. DCM; The results are presented as the mean ± SEM.

To evaluate the effect of CO in DCM, mice were administered a high‐fat diet for a duration of 8 months, with STZ injection occurring at the midpoint of this feeding regimen to induce a model of DCM. Following the administration of STZ, CORM‐2/iCORM‐2 were intraperitoneally administered for a subsequent period of 4 months (Figure 5A). The heart weight to tibia length ratio is an important measure of heart hypertrophy. The DCM mice exhibited a higher ratio of heart weight to tibia length (*p* < 0.05) than that in the control mice (Figure 5B). As shown in Figure 5C, the DCM mice exhibited a higher blood glucose level (*p* < 0.05) than that in the control mice. The weight of control mice increased stably, whereas that of the DCM mice increased faster in the first 4 months and declined sharply to a level lower than that of the DCM mice after STZ injection (Figure 5D). Disorganized myocardial tissue structure, increased collagen deposition, reduced mitochondrial injury scores (Flameng scores), and reduced ventricular ejection fraction and shortening fraction were observed in DCM mice but not in control mice (Figure 5E–I). CORM‐2 treatment did not affect the ratio of heart weight to tibia length, blood glucose, or body weight but ameliorated cardiac mitochondrial injury, fibrosis, fractional shortening, and ejection fraction (*p* < 0.05) in DCM mice. However, the iCORM‐2 treatment showed no effect at all in these mice.

### 3.6 CORM‐2 Treatment Decreased IL‐33 and ST2 Expression and Alleviated Heart Pyroptosis in DCM Mice

**Figure 6:**
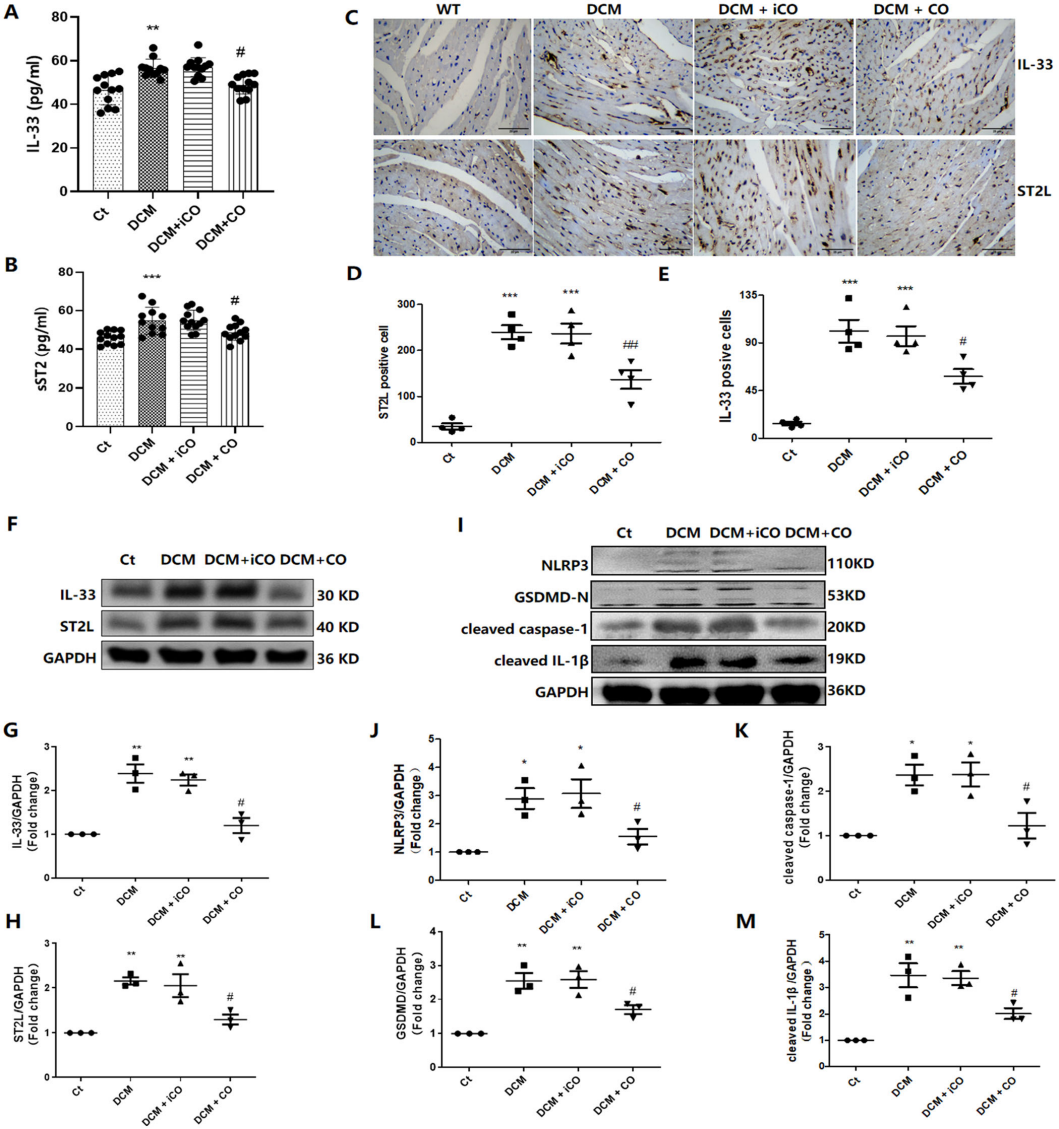
CORM‐2 treatment reduced the levels of IL‐33 and ST2 in the mice with DCM. (A and B) The serum levels of IL‐33 and sST2 was measured with ELISA kits (*n* = 11–12). (C–E) Representative images of heart tissue stained with IL‐33 and ST2L and the statistical data of positive expression (400 ×, *n* = 4). (F–H) Western blot was performed to measure the protein expression of IL‐33 and ST2L in heart tissue (*n* = 3). (I–M) The protein expression and fold changes of NLRP3, GSDMD‐N, cleaved‐caspase‐1, and cleaved IL‐1β (*n* = 3). *p < 0.05 vs. the Ct; **p < 0.01 vs. Ct; ***p < 0.001 vs. Ct; ^#^ *p* < 0.05 vs. DCM; The results are presented as the mean ± SEM.

Next, we investigate the effects of CORM‐2 on the IL‐33 and ST2 levels in DCM mice. As shown in Figure 6A–F, compared with those in the control mice, the serum levels of IL‐33 and sST2 and the myocardial levels of IL‐33 and ST2L increased significantly in the DCM mice. IL‐33 and ST2 levels in both serum and heart tissue were significantly reduced in DCM mice after CORM‐2 treatment (*p* < 0.05). However, the iCORM‐2 treatment showed no significant effect on either IL‐33 or ST2 levels in DCM mice. As DCM mice exhibit pyroptosis, we evaluated the effect of CO on pyroptosis markers in these mice. Compared with those in the controls, the expression of pyroptosis‐related proteins was increased in DCM mice (Figure 6I–M). Interestingly, CORM‐2 treatment reduced the protein expression of pyroptosis‐related proteins including NLRP3, GSDMD‐N, cleaved caspase‐1, and cleaved IL‐1β (*p* < 0.05) (Figure 6I–M). However, the iCORM‐2 treatment did not alter these markers in DCM mice.

## 4 Discussion

Pyroptosis is a highly inflammatory form of programmed cell death that plays a significant role in the progression of DCM progression [29, 30]. It is a component of many diseases, such as chronic kidney disease and atherosclerosis, and its degree becomes more pronounced with disease progression. Targeting pyroptosis in DCM is considered a new promising therapeutic strategy [31]. We showed that IL‐33 is upregulated in DCM, and this is associated with cardiomyocyte pyroptosis. Concurrently, CO treatment inhibited the IL‐33/ST2L axis, thereby, improving myocardial injury and reducing cardiomyocyte pyroptosis. Our results indicated that CO treatment may alleviate cardiomyocyte pyroptosis partially by inhibiting the IL‐33/ST2L axis.

DCM is considered a chronic inflammatory disease. As damage‐associated molecular patterns, NLRP3 are important markers in DCM [13] and can be activated by dead cardiomyocytes. Both hyperglycemia and hyperlipidemia induce an increased production of advanced glycation end products (AGEs) and reactive oxygen species (ROS). Both AGEs and ROS can trigger the activation of NLRP3 to induce pyroptosis. Additionally, endoplasmic reticulum stress and Ca^2+^ homeostasis imbalance can promote the NLRP3 inflammasome assembly, which consequently activates pyroptosis [32]. Drugs such as metformin and atorvastatin can effectively alleviate DCM by inhibiting cardiomyocyte pyroptosis [29, 30].

As a member of the IL‐1 family, IL‐33 can induce inflammatory responses [33]. According to Chen et al. [34], IL‐33 is released extracellularly in NLRP12 and NLRC4 inflammasome‐induced epithelial cell pyroptosis. IL‐33 contributes to the positive feedback loop through the NLRP12/NLRC4‐pyroptosis axis. Therefore, neutralizing IL‐33 may help to block this amplification cascade [34]. In addition, IL‐33 triggers inflammasome activation and induces HK‐2 cell pyroptosis [35]. IL‐33 can increase macrophage pyroptosis in mice with sepsis by activating the NF‐kB/p38MAPK signaling pathway [36]. Taken together, these results indicate that IL‐33 may contribute to cell pyroptosis and induce injury. IL‐33 expression increases during cardiac inflammation as well as after cardiac injury [37]. In the present study, IL‐33 and its receptor ST2L were upregulated in HG‐induced cardiomyocytes and promoted cardiomyocyte pyroptosis. Our conclusion supports results from a previous study that revealed that IL‐33 upregulation may promote cell pyroptosis.

CO is a gaseous signaling molecule that demonstrates pharmacological effects similar to those of nitric oxide [38, 39]. Various forms of CO donors have been developed to explore the effects of CO [40, 41]. Among the CO donors, carbonyl complexes with either a transition metal ion or borane (termed CORMs) have played the most prominent roles [42]. CORM‐2 is a carbonyl complex with a transition metal, and HO‐1 is the primary source of circulating CO. HO‐1/CO has anti‐inflammatory and antioxidant functions, which are important in the protective roles of HO‐1/CO in multiple injury models. HO‐1 metabolizes heme to generate CO, biliverdin, and iron. In addition, HO‐1 overexpression exacerbates heart failure with aging and pressure overload [43]. Also, HO‐1 activation could induce iron accumulation in mitochondria and cause cardiomyocyte ferroptosis in doxorubicin‐induced cardiotoxicity [44]. As HO‐1 overexpression potentially damages cardiomyocytes, especially in chronic disease, and endogenous HO‐1/CO induction also produces by‐products that could decrease treatment specificity, we incorporated CORM‐2 in our experimental design.

CO has both cardioprotective and anti‐inflammatory effects. Accordingly, CORM‐3 alleviates cardiac impairment by inhibiting the binding of NLRP3 protein to ASC [45]. Our previous results showed that CORM‐2 effectively alleviates alcohol‐induced liver inflammatory stress [46]. CORM‐3 also ameliorates neuronal pyroptosis and promotes neurological recovery via the sGC‐cGMP pathway in rats following hemorrhagic shock and resuscitation [24]. Additionally, CO attenuates neuronal cell pyroptosis in rats with traumatic brain injury through the protein kinase G/ERK1/2 signaling pathway [21]. Our results support these previous findings, as we observed that CORM‐2 treatment alleviated heart injury and cardiomyocyte pyroptosis by downregulating the IL‐33/ST2L axis in DCM, whereas iCORM‐2 demonstrated no effects. These results indicate that CO ameliorates cardiomyocyte pyroptosis in DCM by inhibiting the IL‐33/ST2L axis.

CORM‐3 attenuates myocardial injury by regulating Ca^2+^ levels in the tissue [47]. Interestingly, Ca^2+^ influx is reportedly accompanied by an increase in IL‐33 production, whereas Ca^2+^ blockade inhibits LPS‐induced IL‐33 production in macrophages [48]. Additionally, increased intracellular Ca^2+^ has been found to induce IL‐33 secretion, in human bronchial epithelial cells treated with mold allergens [49]. Moreover, it was revealed that the activation of the Ca^2+^‐dependent pathway contributes to IL‐33 release [50]. These findings suggest that CO might inhibit IL‐33 by regulating Ca^2+^ levels. Additionally, CO treatment can reduce IL‐33 expression and alleviate lung injury by decreasing Toll‐like receptor 4 levels [51]. Taken together, these findings indicate that CORM‐2 may play a protective role by reducing IL‐33 levels by influencing various pathways. Our results suggest that CORM‐2 treatment plays a protective role in DCM by downregulating the IL‐33/ST2L axis.

However, this study has several limitations. First, the exact mechanism by which CORM‐2 inhibits the IL‐33/ST2L axis is not yet fully understood and requires further elucidation. Second, the role of IL‐33 in mediating pyroptosis in cardiomyocytes exposed to high glucose conditions remains insufficiently comprehended, warranting future investigative efforts. Third, we used only male mice, further studies involving female mice are required. Fourth, future research should incorporate the stimulation of cells with both high glucose and high fat. Additionally, significant species differences between rodents and humans may influence treatment response. Thus, it remains essential to employ humanized models or multi‐omics approaches to facilitate the clinical translation of CO treatment in the future.

## 5 Conclusions

Carbon monoxide (CO), often referred to as a “silent killer,” has been increasingly recognized for its therapeutic potential in cardiovascular diseases. However, to date, there is a lack of research regarding its impact on DCM. In this study, our results indicate that the upregulation of the IL‐33/ST2L axis exerts a pyroptosis‐promoting effect in DCM, and CORM‐2 treatment can ameliorate cardiomyocyte pyroptosis, in part, by downregulating the IL‐33/ST2L axis in DCM mice. The findings provide novel insights into the prevention and treatment of DCM.

## Author Contributions

**Chunjie Jiang:** funding acquisition, writing – original draft. **Ping Zhu:** writing – original draft. **Ping Yao:** conceptualization. **Xiaojun Bi:** investigation, writing – review and editing. **Chao Gao:** conceptualization, funding acquisition, writing – review and editing.

## Consent

The authors have nothing to report.

## Conflicts of Interest

The authors declare no conflicts of interest.

## Data Availability

The data sets used and analyzed are available from the corresponding author upon reasonable request.
